# Cardiac rehabilitation - The answer for the second chance

**DOI:** 10.1016/j.ahjo.2022.100108

**Published:** 2022-03-08

**Authors:** Sarah Alexander, Shannon Li, Melissa Tracy

**Affiliations:** a Community Care Network, Inc., Munster, IN, United States of America; b RUSH University Medical Center, Chicago, IL, United States of America

**Keywords:** Cardiac rehabilitation, Women, Female patients, CVD trials, Under representation

## Abstract

In the United States and worldwide, the leading cause of death in females is cardiovascular disease (CVD). However, compared to males, females have overall higher mortality rates, especially within the first few years of having an acute myocardial infarction (AMI). Despite the increased awareness of CVD in females and established benefits of cardiac rehabilitation (CR) programs, there is still delayed initiation of care, under-recognition of atypical presentations of angina in females, under referral of females to CR, and under-representation of females in CVD trials. In this paper, we will investigate the barriers to female participation in CR, explore the fundamental differences in physiology between males and females, and current limitations in CVD trials where females are under-represented. Finally, we aim to provide potential methods to increase enrollment of females in CR and CR related trials.

## 1 Introduction

Cardiovascular disease (CVD) remains the leading cause of death in females worldwide.^1^ In the United States alone, approximately 60 million females are affected by CVD annually including coronary artery disease (CAD), cerebrovascular accident (CVA), heart failure (HF) and peripheral artery disease (PAD). Based on the 2020 AHA Heart Disease and Stroke Statistics 2020 Update, of the 6.6 million patients diagnosed with acute coronary syndrome (ACS), 2.6 million were females.^1^ When compared to males, regardless of age, more females will die within the first year of acute myocardial infarction (AMI) (26% females vs 19% males), and more females will die of HF or have a CVA within the first 5 years of an AMI (47% vs 36%).[1], [2] These are often a consequence of delayed initiation in cardiac catheterization and guideline directed therapy in females who tend to have atypical anginal presentations.[3], [4], [5] Encouragingly, over the past decade, there has been a drastic decline in mortality rates for females, as a result of increased awareness of the atypical presentation and a better understanding of mechanisms of ACS in females, including microvascular dysfunction and plaque erosion.^2^ There has also been increased recognition of less frequently encountered conditions that tend to manifest more in females including spontaneous coronary artery dissection, Takotsubo syndrome, and coronary vasospasm, all of which still remain largely underdiagnosed.^2^ However, despite the increased awareness of CVD in females, the incidence of AMI continues to rise due to the growing prevalence of obesity, metabolic syndrome, hypertension, hyperlipidemia, tobacco use, and chronic kidney disease.^1^ From a social standpoint, females are more likely than males to bear a caregiver role while also contributing financially to the household, which can result in higher degree of depression, anxiety, and stress.^6^ Such factors have been shown to increase the burden and affect the recovery of CVD in females.[2], [7]

CR is an important feature of CVD treatment that needs increased study and participation of females. As a Cardiology community, we are increasingly aware of male/female differences in CVD. The factors underlying these differences involve both biological and sociocultural characteristics and need to be better understood and defined.

In this review, we aim to address the roles of cardiac rehabilitation (CR) and CR trials in CVD treatment as it relates to females.

## 2 Sex and gender

Sex refers to the biological and physiological characteristics corresponding to the karyotype present at birth (46XX, 46XY). Gender refers to social, cultural, and environmental characteristics that influence the identity of a person (woman, man, or non-binary).[10], [11] Both sex and gender should be evaluated when discussing barriers to female/women participation in CR and CR trials. For the purposes of our review, we will focus on sex/female related characteristics.

## 3 Importance of cardiac rehabilitation

Given the burden of CVD in females and despite improved mortality rates, there needs to be continued focus on treatment and recovery from CVD. Among proven treatment approaches is CR, which is an essential part of post MI care and is a Class IA recommendation per the AHA guidelines.[12], [13], [14] CR initially was focused on providing exercise training to almost exclusively middle-class men younger than 65 years-of-age with uncomplicated MI or coronary bypass surgery.^14^ However, we now know that the benefits of CR extend to the general population as well and has been shown to decrease cardiac mortality and hospitalizations within the first year after MI.^14^ Reduced hospitalizations and mortality due to CR are also seen in patients with chronic stable angina and heart transplants.^14^ In 2006 and 2014, CR qualifying diagnoses were expanded to include patients with heart valve replacement/repair, percutaneous coronary interventions, PAD and chronic systolic HF. [14], [15], [16], [17]

**Fig. 1:**
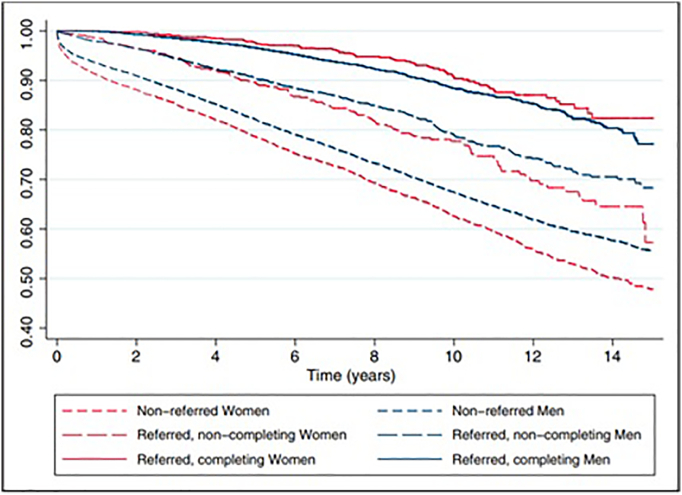
Survival stratified by referral, attendance, and sex.

Despite the proven benefits of CR, females are less likely to be referred to CR and remain under-represented in CR trials compared to males.[6], [7], [8], [9] Females remain under-enrolled in CR even though some programs have offered female-only CR.[16], [18], [19] Moreover, enrollment into CR programs does not guarantee completion of the program. Based on the EUROASPIRE III survey, which was conducted at 76 CR centers in 22 European countries, only 36.5% of the 8845 eligible patients participated in CR.[16], [20] Higher participation rates were noted in younger patients, male sex, and higher educational level.[8], [21] In the United States, only 12.2% of eligible patients participated in CR based on one of the largest studies on CR utilization among 601,099 Medicare beneficiaries.^19^ A large Canadian cohort study by Stone et al. involving 25,958 patients (24.6% females) showed that females who had been to CR and had completed the program had the greatest reduction in mortality or survival benefit (HR 0.36, 95% CI 0.28, 0.45) while females who were not referred to CR had the greatest mortality.^22^ Furthermore, females experienced a relative mortality benefit compared to males (HR 0.51, 95% CI 0.46, 0.56) (Fig. 1).[7], [22]

## 4 Limitations to female participation in cardiac rehabilitation

The benefits of CR have been clearly established. Yet, female patients are still not participating. This is a multifactorial problem and can be considered at three different levels: the health systems, provider, and patient levels.

At the health systems level, females are less likely to be referred to CR than males simply due to a lack of referral and a lack of exposure to CR while in the hospital recovering from a cardiac event. A meta-analysis of 19 observational studies examining differences in referral rates by sex among over 240,000 participants found that females were 32% less likely to be referred to CR compared with males despite similar eligibility.^9^ At the provider level, healthcare providers (HCP) may not appreciate the impact of CVD on females or the benefits of CR; therefore, albeit unintentionally, not discuss CR with their female patients.^7^ Studies have shown that females tend to have CVD events at older ages compared to males and to have more comorbidities upon presentation with a cardiac event. There may be an HCP referral bias in that providers feel that older patients with more comorbidities may not be able to physically engage in CR and/or are less likely to benefit.^18^ In addition, HCPs may not have an understanding of the CR program and what it entails.^18^ Therefore, their knowledge gap results in a withholding of optimal medical therapy. Finally, at the patient level, notable factors preventing females from participating in CR include being single/unmarried, uninsured, socioeconomically disadvantaged, obese, elderly, nonwhite, less educated, and suffering from concurrent psychiatric disorders such as depression.[8], [15], [20], [23] Personal reasons include balancing both family caregiver role along with work responsibilities; logistical reasons include transportation limitations, perception of the objective of CR, inconvenient timing, lack of flexibility, and financial reasons.[6], [8], [21], [48] Single/unmarried females may have less support from within and outside their home in their recovery after a CV event.^21^ It is essential to provide follow-up support and consistent information from care providers, CR, and peer support programs to female patients to encourage ongoing engagement and participation.^24^ The Centers for Disease Control and Prevention estimates that 58% of all caregivers in the United States are females.[25], [26] Females, for the most part, are ready to advocate for their loved ones, yet place their own advocacy in the queue.^24^

## 5 Under-representation of females in cardiac rehabilitation trials

Recognizing the barriers to female participation in CR should be driven by data and clinical research trials. We need to understand how current data are obtained and how females are (under)represented in CVD clinical trials.

Due to the historic under-representation of females in clinical research, Congress approved the NIH Revitalization Act of 1993, which directed the NIH to establish guidelines for the inclusion of females and minorities in clinical research. The majority of drug trials are sponsored by pharmaceutical companies which are regulated by the FDA; however, the FDA does not mandate such inclusion as does the NIH.[27], [28] Lindley et al. published a timely review, highlighting the need for more female enrollment in clinical CVD trials, noting that specifically, females are disproportionately affected by CVA, HF with preserved ejection fraction, and myocardial infarction with nonobstructive coronary arteries, yet females constitute only 38% of participants in clinical trials and 33% in CVD trials.^29^ Supervia, et al. conducted a literature search regarding the barriers of female participation in CR. They identified a total of 31 studies assessing the impact of various interventions aimed at improving CR participation: the percentage of female participants in these studies was only 37.37%, with 27 of the 31 studies including less than 50% of females, only six studies reported separate results for males/females, and only 3 studies assessed interventions to improve CR participation exclusively in females.^18^ Finally, Vidal-Almela, et al. recently published a narrative review of factors influencing women's participation in cardiac rehabilitation.^48^ Findings from their review are highlighted in our manuscript for methods to overcome female barriers.

## 6 Sex and gender influences on trial design

Future CR trials should consider both sex and gender related factors. Differing biological factors and socioeconomic factors between sexes not only drive CVD pathophysiology, but also influences female CVD interventions and acceptance of CVD medical options. There are no safety or efficacy reasons for the underrepresentation of females in CR programs. Most CVD trials are under-powered to thoroughly evaluate sex differences and sex related impacts, which result in the extrapolation of therapeutic interventions to the female patient from mostly male participant derived data. There is a need to incorporate sex into the study design, justify any sex-specific exclusion criteria, and perform sex-specific analyses to evaluate efficacy and/or harm in both males and females. Both sexes must be represented proportionate to sex distribution of the disease to improve randomized control trial (RCT) generalizability and ensure a sufficient sample size to investigate sex-specific treatment effect and safety. While these criteria are justified for some interventions, they have often been applied broadly and without justification in the context of individual trials.^30^ The Sex and Gender Equity in Research (SAGER) guidelines can be used as a framework to improve the reporting of sex-specific outcomes in clinicals trials.^17^

## 7 Sex related factors

When CVD trials are hypothesized, developed, and implemented, it is imperative to take into consideration of the different biological effects of testosterone and estrogen (whether it be endogenous, synthetic, *peri*/postpartum, or changes in estrogen level) on the vascular system. These hormonal factors, which are clearly different between the sexes, will impact CVD outcomes and treatments. The decision to exclude pregnant or lactating females from RCTs should involve careful consideration of the intervention and comparator groups, with risk assessment informed by biological plausibility and preceding research data.^30^ It is crucial to include pregnant and lactating females in CVD trials to improve CVD care for both females and their children. Many interventions can be safely studied within the monitored setting of an RCT, which typically involves closer follow-up than in routine clinical settings.^30^

Physiologically, the distribution of adipose tissue and the amount of skeletal muscle also influence how males and females compensate and respond to exercise deconditioning. Females have increased subcutaneous and intramuscular fat but reduced skeletal muscle, which can result in increased inflammation, insulin resistance, and myostatin levels (a protein produced and released by myocytes which inhibits muscle growth).^31^ Lifestyle interventions including exercise and weight loss have been shown to improve exercise capacity and quality of life due to favorable pleiotropic effects on both the non-cardiac and cardiac systems, primarily through improvements in systemic inflammation, adiposity, and peripheral oxygen utilization in skeletal muscle.^31^ These hormonal factors can have an array of influences and must be taken into account when patients are being counseled, monitored, and treated by their Cardiac specialist. Providing HCP with evidence-based guidelines to help them understand the pathophysiology and the proven benefits of CR programs is essential in providing safe healthcare, as a lack of evidence-based care may result in harm.

Entirely separate recommendations for males and females are not required as long as the published material includes recommended modifications and adjustments based on sex and reproductive status. Future guidelines should highlight knowledge gaps and demonstrated sex differences. Female enrollment in clinical trials will help improve informed decisions about treatments based on high-quality efficacy and safety data.^32^ Guidelines, education through meetings/webinars, and medical practice requirements have the potential to translate knowledge to action and improve care for females in clinical settings.

**Fig. 2:**
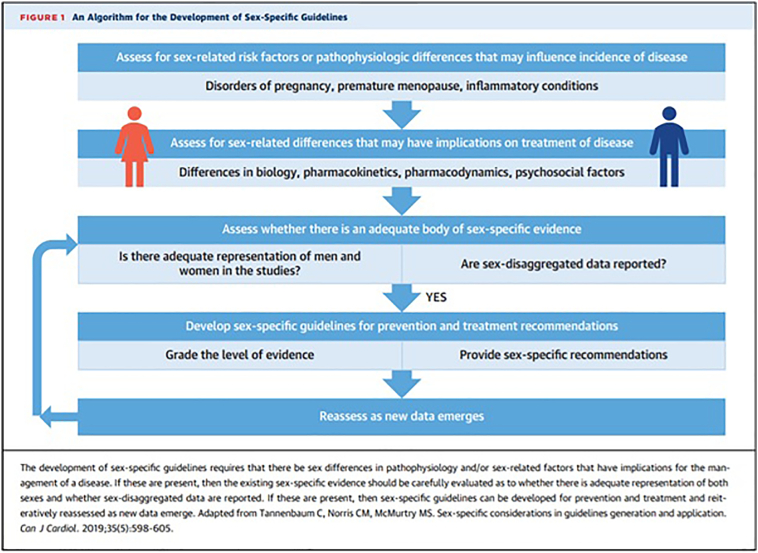
Sample algorithm for developing sex-specific guidelines.^28^

Several published and accepted guidelines for the treatment of CVD have suggested that females be treated with the same therapies as males—a unisex approach for a gender impartial disease. Fig. 2 is an algorithm for the development of sex-specific guidelines, which was first suggested by Tannenbaum and based on a systematic appraisal of sex-related risk factors and pathophysiology which may alter treatments based on evidence: “*The development of sex-specific guidelines requires that there be sex differences in pathophysiology and/or sex-related factors that have implications for the management of a disease. If these are present, then the existing sex-specific evidence should be carefully evaluated as to whether there is adequate representation of both sexes and whether sex-disaggregated data are reported. If these are present, then sex-specific guidelines can be developed for prevention and treatment and reiteratively reassessed as new data emerge. In the meantime, there is value in having trials report data disaggregated according to sex, even if underpowered, to enable pooling of sex-specific data during meta-analysis*”.^33^

(Permission obtained from Defilippis JACC, 2021) JACC Vol 78, No 2. 2021 pp 189-192.

## 8 Increasing female participation in cardiac rehab and cardiac rehab trials

Given the limitations on female participation in CR, we offer the following approaches to increase female enrollment and participation in CR and CR related CV trials.

### 8.1 Emphasize Phase I CR as part of inpatient cardiovascular care-knowledge is power!

The incorporation of structured Phase I CR back into the post CVD care is needed. After a CV event, patients are vulnerable and need guidance. Positive endorsement of CR from our healthcare team members will result in patients' embracement and participation of CR into their immediate and long term lives.[15], [34] A multidisciplinary team approach including nurses, house staff, faculty, physical therapists, nutritionists, social workers, and home health care workers is vital to helping patients understand the importance and long-term objective of CR, which ultimately extends beyond their current hospital stay.

### 8.2 Provide automatic referrals to outpatient Phase II CR and incorporation of patient care liaisons to facilitate CR enrollment addressing cost, transportation issues, and other patient related barriers (see Fig. 3, permission obtained)

It has been well established that a lack of referral is the primary limiting factor in CR participation.^34^ This must change. Having CR liaisons to help facilitate the continuum of care from inpatient to outpatient are imperative, especially for patients discharged to skilled nursing facilities.

**Fig. 3:**
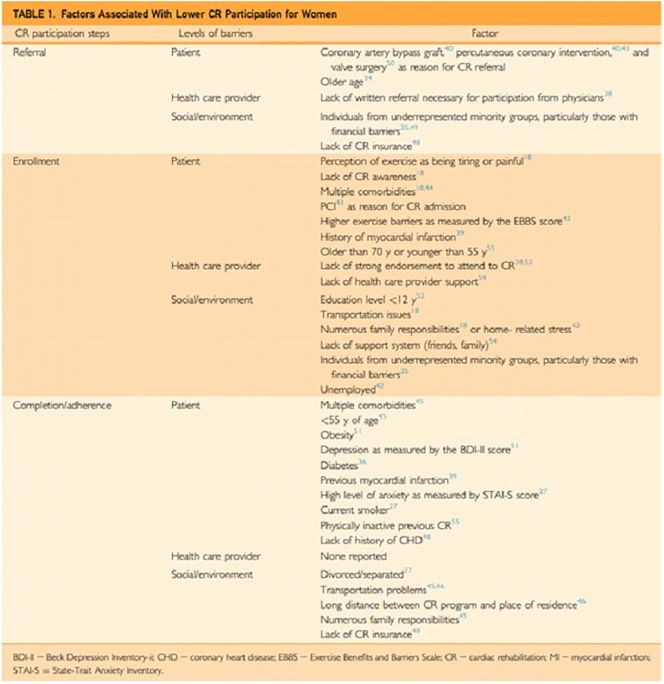
Mayo.

### 8.3 Increase use of Home-Based Cardiac Rehab (HBCR)/virtual programs and other Cardiac Rehab Alternatives

Future studies exploring CR options, besides the traditional center-based CR (CBCR) for female patients with CVD, are warranted. To attain the CR participation rate goal of 70% by 2022 set by the Million Hearts Initiative,[35], [36] CR programs will need to be expanded beyond the confines of CBCR. An effective CR option must include the key components of a comprehensive CR program: exercise, nutrition, risk factor modification, psycho-social support, and education coupled with non-inferior outcomes. The utility and efficacy of high intensity interval training (HIIT), HBCR programs, and female-only exercise sessions are areas of focus that could have a positive influence on increasing both the enrollment of female patients and completion of CR programs. HIIT is an example program that could address the time restraints faced by females. Unfortunately, current studies comparing the health and psychological benefits of HIIT vs continuous exercise are predominantly based on male participants.^37^ The amount of time required for the recommended continuous exercise of moderate-to-vigorous intensity of at least 150 min per week is an important limiting factor to acknowledge, as it is associated with a higher dropout rate when compared to HIIT, which requires a shorter time commitment.^37^ If CR programs could be tailored to better accommodate the time restraints of females, this might help improve their participation.^48^ Adjusting the time (i.e evening and weekend classes) and location of CR classes would also facilitate greater female participation.[38], [48] Utilizing community centers rather than a hospital setting can address the need of “community” and “recovery” for our female patients with more of an emphasis on well-being and health, rather than illness.[38], [48]

As a result of the COVID-19 pandemic, CR facilities closed down, leaving a gap in CV care. Alternative options to CBCR have been studied and proposed for years. These include hybrid, home based/virtual (HBCR), and community-based CR programs. The Hybrid CR program is a combination of telehealth, on-demand, and in-person sessions, which combines the best of both worlds. HBCR programs include all of the core components of CR delivered via virtual face-to-face visits with a dietician, physical therapist, and medical staff along with on-demand lifestyle modification videos with and without monitoring. Community-based CR programs incorporate community sites/centers to deliver the exercise portion without monitoring. It is important to note that the HBCR has been proposed and endorsed by the American Association of Cardiovascular and Pulmonary Rehabilitation (AACVPR) and is defined as systematic, comprehensive, and personalized services that involve medical evaluation, prescribed exercise, CV risk factor modification, patient education and behavioral activation/counseling that are delivered mostly or entirely outside of the traditional CBCR setting.^39^

HBCR has long been proposed as a viable and effective option for patients after a CV event. DeBusk et al. published a case management study, MultiFit Trial, looking specifically at HBCR compared to usual care for patients after a MI.^40^ A total of 585 patients were enrolled at 5 different centers in California, and those who completed HBCR had statistically significant differences in the reduction of LDL cholesterol, greater success at smoking cessation, and improved exercise capacity.^40^ Published in New England Journal of Medicine (NEJM) Catalyst 2019 was a well-structured program utilizing virtual CR and Samsung technology.^41^ Compared to traditional on-site CR, completion rates for the virtual program were improved by 74% and total referrals to CR increased 44.5%.^41^ Furthermore, there was reduced hospitalization rates of <2% for patients post MI and stent with the national average readmission rate of 10–15%.^41^ However, how and when to implement a program has been challenging, given a paradigm shift from the standard of care.

HBCR has provided a necessary option for patients during the COVID-19 pandemic and also serves a role for those interested in CR, but are limited in their ability to participate. HBCR allows more flexibility and provides a safe and effective CR alternative, 24-h-a-day, 7-days- a-week, in the privacy and safety of home. HBCR is endorsed by the ACC, the American Heart Association (AHA), and AACVPR. The ACC published an Expert Analysis stating that the data show that virtual CR is an equivalent alternative to center-based CR.^42^ Under the Public Health Emergency related to COVID-19, virtual CR will continue throughout the entirety of the pandemic with a mechanism for reimbursement secured. The barrier for the post COVID era will be reimbursement.^20^ Expansion of CR resources will need to be accompanied by close monitoring of program quality using standardized measures from CBCR. Furthermore, the “readiness” for our patients to utilize technology must be assessed. Most patients have access to a cell phone, but may not have the experience to incorporate their device into their healthcare. Patient navigators are imperative to assist our patients, especially the older patients who account for 25% of physician office visits.^43^ Policies need to be developed to help bridge the digital divide. Research identifying the reason(s) why HBCR option was/was not chosen by a patient, the demographics of the patients attending HBCR, and clinical outcomes of patients participating in CBCR vs HBCR should be evaluated. These alternative options can ultimately result in lifelong positive and preventive implications for our female patients and their families/communities.

### 8.4 Encourage females to participate in CVD research

Along with limited female participation in CR programs, females are under-represented in CR related research as well. Due diligence to address these barriers must be meticulously and rigorously addressed in future clinical trials and published guidelines. Federal agencies and pharmaceutical companies should not provide funding for research without an appropriate number of participants, distinguished by sex and ratioed to the disease being investigated. This hopefully will result in increased number of participants and more impactful findings.

Females need to be encouraged to become research participants. This will provide new learning and improvement in treatment tailored to female patients.^24^ Females may perceive the trials to be dangerous, experimental, and/or not clearly understand the implications of the trial.^44^ Ensuring participants are aware that clinical trials are conducted with methodological rigor and are closely monitored for safety may alleviate fears and increase enrollment.^44^ Targeting marketing efforts at local places of worship and beauty salons and using social media strategies may also improve awareness. Leveraging web-based participation may help enroll females with caretaking responsibilities and geographic or transportation barriers.^29^

Moving forward, we must acknowledge the progress that has been made. Recently published in the NEJM, Physical Rehabilitation for Older Patients Hospitalized for Heart Failure, the investigators sampled an equal number of female patients, and provided sex specific data in regards to the impact of their intervention.^45^ Also, the recently published AHA 2021 Guidelines for the Evaluation and Treatment of Chest Pain highlights the over classification of chest pain in females as non-cardiac, despite that most females are ultimately diagnosed with ischemic cardiac chest pain.^46^ Our Cardiology Community should be striving for improved healthcare of our female patients based on increased recognition and acknowledgement of these atypical presentations and under-representation of females in CR and CV related trials.

### 8.5 Provide social support to females after a CV event - “It's O.K. not to be O.K.” Naomi Osaka^47^

Studies have shown that females desire social and peer-to-peer interactions for their CV recovery.^48^ Providing a social network for females will increase their participation and adherence to CR.[22], [24], [38], [48] Compared to males, females benefit more in their CVD recovery from support groups that include shared peer experiences and individual stories, which help them to re-create their self-image and perspective on life to a new “normal” after a CV event.[22], [24], [48] In addition, females would benefit from improved self-education on their health conditions, which would help them to become better self-advocates when talking to their HCPs.

### 8.6 Involve HCPs in the treatment plan and educate HCPs about CR

Female patients have an established relationship with their HCP, therefore, an endorsement from a trusted HCP would help encourage patients to enroll in CR and CVD trials. Our HCPs must be encouraged through CME requirements and NBME qualifications to narrow their knowledge gap on proven CVD interventions. Primum non nocere, “First do no harm”.

## 9 Conclusions

In spite of the overwhelming CVD burden on our female patients, the therapeutic options are mainly extrapolated from CVD trials based on mostly white male participants. CVD pathophysiology, treatments, and outcomes can require sex specific alterations. Females have unique biological, hormonal, and societal factors which have important impacts on their CVD health. These important sex and gender differences must be taken-into-account. The only way to appropriately achieve this goal is by increasing the number of female participants in CVD trials. CVD trials should not be allowed, without clear explanation and documentation, to under-power a trial which could impact treatment implications for both sexes. Recruitment of females into CVD trials should be required by funding sources, review boards, regulatory agencies, and medical journals.^30^ Alleviating fears and apprehensions regarding participation in CVD trials needs to be strategically fostered. Equalizing the playing field of research investigators, and diversifying the cardiology work force to better represent our patient demographics, are imperative for female inclusion and participation, which ultimately would result in health benefits for all patients.^30^ Detailing who interviewed the patient and why the patient declined participation may assist us in finding additional avenues we need to address. Furthermore, compared to the traditional and more time consuming in-person participation, alternative technological methods of incorporating female participation into trials should be explored. Removing sex bias and working toward empowering and educating our female patients, regarding their individual risks, medical options, and alternative options to medical care, will result in improved CVD outcomes.

## Abbreviations

AMI: acute myocardial infarction
CAD: coronary artery disease
CBCR: center based cardiac rehabilitation
CVA: cerebrovascular accident
CVD: cardiovascular disease
CR: cardiac rehabilitation
HBCR: home based(/virtual) cardiac rehabilitation
HCP: healthcare provider
HF: heart failure
HIIT: high intensity interval training
RCT: randomized controlled trial

## Declaration of competing interest

The authors declare the following financial interests/personal relationships which may be considered as potential competing interests: Melissa Tracy is a consultant for Virtual Health Partners (VHP).
